# Endothelial TREM‐1 receptor regulates the blood–brain barrier integrity after intracerebral hemorrhage in mice via SYK/β‐catenin signaling

**DOI:** 10.1111/cns.14255

**Published:** 2023-05-11

**Authors:** Yonglin Xie, Wei He, Li Ma, Reng Ren, Shuxu Yang, Qin Lu

**Affiliations:** ^1^ Department of Emergency, Sir Run Run Shaw Hospital Zhejiang University, School of Medicine Hangzhou China; ^2^ Department of Pharmacy, Second Affiliated Hospital Zhejiang University, School of Medicine Hangzhou China; ^3^ Department of Neurosurgery, Sir Run Run Shaw Hospital Zhejiang University School of Medicine Hangzhou China; ^4^ Department of Neurointensive Care Unit, The Second Affiliated Hospital Zhejiang University, School of Medicine Hangzhou China

**Keywords:** BBB permeability, brain edema, intracerebral hemorrhage, SYK/β‐catenin signaling, triggering receptor expressed on myeloid cells 1

## Abstract

**Background:**

Intracerebral hemorrhage (ICH) is a high mortality and disability stroke subtype. Destruction of the blood–brain barrier (BBB) is a crucial contributor to brain edema and neurological deficit after ICH. Triggering receptor expressed on myeloid cells 1 (TREM‐1) has been reported to be expressed in endothelial cells, but its role in ICH remains unclear. This study aims to evaluate the role of TREM‐1 on BBB permeability after ICH in mice.

**Methods:**

Two hundred and forty‐two CD1 mice were used in this study. The ICH model was established by collagenase injection. LP17 was administered intranasally at 2 or 8 h after ICH to inhibit TREM‐1. To explore the underlying mechanism, SYK activation CRISPR was administered intracerebroventricularly with LP17, and Anti‐mouse TREM‐1 rat IgG2a (a specific TREM‐1 agonist) was injected intracerebroventricularly with R406 (a specific SYK inhibitor) intraperitoneally. Neurobehavioral outcome, brain water content, BBB permeability, and protein expression were evaluated.

**Results:**

The expression level of the TREM‐1 receptor increased rapidly as early as 6 h after ICH, and it was mainly expressed on the endotheliocytes in the neurovascular unit. Early and delayed administration of LP17 significantly decreased brain edema and improved neurobehavioral outcomes at 24 h after ICH. LP17 reduced the BBB permeability by increasing β‐catenin, claudin‐5 and ZO‐1 expression. Furthermore, SYK activation CRISPR abolished the beneficial effect of LP17 on the expression of the above junction molecules. Meanwhile, R406 reversed the impact of the TREM‐1 activator on the downregulation of β‐catenin, claudin‐5 and ZO‐1 expression.

**Conclusions:**

This study demonstrated that TREM‐1 deteriorated BBB permeability via modulating the expression of interendothelial junction molecules after ICH, and this regulation is partly mediated by the SYK/β‐catenin signaling pathway.

## Graphical abstract

Blood–brain barrier broken is a crucial pathological process in intracerebral hemorrhage (ICH), which leads to seconding brain injury. Our study discovered that triggering receptor expressed on myeloid cells 1 (TREM‐1) activated in endothelial cells after ICH. Then, TREM‐1 activated the SYK/β‐catenin signaling pathway and decreased the expression of junction molecules among endotheliocytes. Thus, TREM‐1 can be a potential therapeutic target.

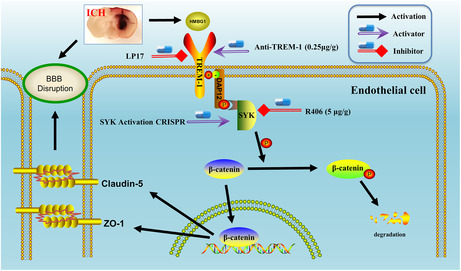

## 1 INTRODUCTION

Intracerebral hemorrhage (ICH), a severe stroke subtype with high morbidity and mortality, accounts for 15%–20% of all strokes.^1^ As one of the critical pathological processes in second brain injury after ICH, disruption of the blood–brain barrier (BBB) integrity results in perihematomal cerebral edema formation and peripheral inflammatory factors infiltration and contributes to high mortality and poor outcome of ICH.^2^ Therefore, inhibition of BBB disruption can be a therapeutic target for ICH patients.

Triggering receptor expressed on myeloid cells 1 (TREM‐1) is an immune‐receptor expressed on the surface of myeloid cells.^3^ Although the pathophysiological role of TREM‐1 was first identified during infectious diseases, increasing studies suggested it participated in no‐infectious disorders.^4^ Furthermore, TREM‐1 was also found expressed on the surface of endothelial cells and regulated the vascular function recently.^5^ However, whether TREM‐1 medicated the disruption of BBB integrity remained unclear.

Activated TREM‐1 will provide a docking site for Spleen Tyrosine Kinase (SYK) via DAP12, then SYK will recruit and phosphorylate various signals.^4^, ^6^ β‐catenin, regarded as a critical signal transducer that regulates gene expression of endothelial junctions,^7^ was also reported phosphorylated by SYK in recent studies.^8^ Inhibition or phosphorylation of β‐catenin decreases junction molecules' expression level and amplifies BBB leakage.^9^

This study hypothesized that the endothelial TREM‐1 receptor could modulate blood–brain barrier integrity by regulating the expression of junction molecules after intracerebral hemorrhage in mice. This effect was mediated through the SYK/β‐catenin signaling pathway.

## 2 METHOD

### 2.1 Animals

Total 242 adults male CD1 mice were used as listed in Appendix S1. All mice were housed in a 12‐h light/dark cycle room with temperature and humidity control. Animals had free access to food and water. All the experimental procedures were approved by the Institutional Animal Care and Use Committee at Sir Run Run Shaw Hospital.

### 2.2 Study design

As shown in Appendix S1, five parts of experiments were designed and all mice were randomly assigned.

#### 2.2.1 Experiment 1

To explore the time‐course expression of TREM‐1 in collagenase‐induced ICH mice. Six groups were set randomly (six mice per group): sham, 6 h, 12 h, 24 h, 72 h and 7 days after ICH, and western blot was used to detect the expression level of TREM‐1. Furthermore, two additional sham mice and two ICH mice at 24 h were used to assess neurovascular unit localization of TREM‐1 by double immunohistochemistry staining.

#### 2.2.2 Experiment 2

LP17 with a dose of 1.0 μg/g was applied to evaluate the role of TREM‐1 on the outcome of ICH mice at two different time points. Groups were established as follows: sham, ICH, ICH + control peptide, ICH + LP17 (1.0 μg/g), ICH + control peptide‐delayed, and ICH + LP17 (1.0 μg/g)‐delayed. Neurobehavioral tests and brain water content were performed 24 and 72 h after ICH.

#### 2.2.3 Experiment 3

To explore the role of TREM‐1 on BBB permeability, 30 mice were divided into three groups: sham, ICH + control peptide, and ICH + LP17 (1.0 μg/g). Evans blue (EB) test and IgG staining were used to evaluate BBB permeability at 24 h after ICH.

#### 2.2.4 Experiment 4

To further study the long‐term outcome of LP17 treatment mice, 24 (*n* = 8 per group) mice were randomly divided into sham, ICH + control peptide, and ICH + LP17 (1.0 μg/g). Long‐term neurobehavioral experiments and Nissl staining were analyzed.

#### 2.2.5 Experiment 5

To verify the downstream signaling pathway that TREM‐1 regulates BBB permeability after ICH. Two parts of groups were set as follows (six mice per group): Part 1, sham, ICH, ICH + control peptide, ICH + LP17 (1.0 μg/g), ICH + LP17 (1.0 μg/g) + control CRISPR and ICH + LP17 (1.0 μg/g) + SYK activation (ACT) CRISPR; Part 2, sham, ICH, ICH + control IgG, ICH + Anti‐TREM‐1 mAb (0.25 μg/g), ICH + Anti‐TREM‐1 mAb (0.25 μg/g) + DMSO and ICH + Anti‐TREM‐1 mAb (0.25 μg/g) + R406 (5 μg/g). The expression level of downstream proteins and endothelial intercellular adhesion molecules were assessed by western blot.

The information of the above groups was blinded to the researchers who performed surgeries, drug administration, neurobehavioral tests, immunofluorescence staining western blot, Nissl staining, and data analysis.

### 2.3 ICH model

The ICH model was established by injection of bacterial collagenase as previously described.^10^ Mice were anesthetized by intraperitoneal injection with a mixture of ketamine (100 mg/kg) and xylazine (10 mg/kg) (2:1). A 1‐mm cranial burr hole was drilled under a stereotaxic head frame (Kopf Instruments) with the coordinates 0.2 mm posterior and 2.2 mm right lateral to the bregma. Then 0.5 mL sterile phosphate‐buffered saline (PBS) with 0.075 U bacterial collagenase type VII was injected into the right basal ganglia at a rate of 0.167 mL/min under a microinjection pump (Stoelting, MA). The needle was left in place for an additional 5 min, then withdrawn 1 mm per minute. The burr hole was covered with bone wax before the scalp was sutured. Finally, 0.4 mL of normal saline was injected subcutaneously to avoid postsurgical dehydration. Meanwhile, the sham operation was performed following the same protocol without injection.

### 2.4 Drug administration

According to previous studies, a selective inhibitor of TREM‐1 called LP17 (GenScript) with a dose of 1.0 μg/g and a control peptide was synthesized and administered intranasally at 2 h after ICH.^11^ Additionally, LP17 was administered at 8 h after ICH to evaluate the effect of delayed treatment. Anti‐mouse TREM‐1 rat IgG2a (0.25 μg/g; Thermo Fisher Scientific), as a specific TREM‐1 agonist, or control rat IgG2a (Thermo Fisher Scientific), was administered intracerebroventricularly. R406 (5 μg/g; Selleckchem) as an inhibitor of SYK was dissolved in dimethyl sulfoxide (DMSO) and administered intraperitoneally. SYK activate (ACT) CRISPR (Santa Cruz Biotechnology) was used to activate *syk* gene expression. 1 μg ACT CRISPR was given intracerebroventricularly 48 h before the animal model set at a rate of 1 μL/min with a micro‐injection pump. The scrambled CRISPRs (Santa Cruz Biotechnology) were injected with the same protocol.

### 2.5 Intracerebroventricular injection

Intracerebroventricular administration was performed as previously described.^12^ A 10 μL Hamilton syringe was inserted into the left lateral ventricle stereotactically, and the coordinates were given as follows: 0.3 mm posterior, 1.0 mm lateral, and 2.3 mm below the dura. The injection rate was 1 μL/min, and the needle was left in place for an additional 5 min before being slowly withdrawn for about 3 min.

### 2.6 Neurobehavior tests

For short‐term assessments, neurobehavioral outcomes were evaluated at 24 and 72 h after ICH by an independent researcher blinded to the procedure including three tests: modified Garcia test, forelimb placement test and corner turn test. For long‐term evaluations, the rotarod test was performed every week after ICH, and the water maze test was assessed on days 21–25 post‐ICH as previously described.^13^ The details of the tests were also listed in the Appendix S1.

### 2.7 Brain water content

Brain water content was measured by the wet/dry method.^14^ Whole‐brain was collected at 24 h after ICH and separated into the ipsilateral cortex, ipsilateral basal ganglia, contralateral cortex, contralateral basal ganglia and cerebellum. The wet weight of brain tissues was obtained by weighing immediately, and then the tissues were dried at 100°C for 48 h before determining the dry weight. Brain water content was calculated as [(wet weight − dry weight)/wet weight] × 100%.

### 2.8 BBB permeability assessment

As reported, BBB permeability was evaluated by EB dye extravasation assay.^15^ Briefly, 4% EB dye (4 mL/kg, Sigma‐Aldrich) was injected intraperitoneally at 3 h before sacrifice evaluation, and mice were sacrificed 24 h after ICH. The right hemisphere was homogenized and sonicated within 1100 μL PBS and then centrifuged for 30 min at 15,000 *g*. 500 μL supernatant from each sample was mixed with 500 μL 50% trichloroacetic acid overnight at 4°C. After centrifugation, the supernatant was collected, and EB dye extravasation was evaluated at 610 nm and quantified according to a standard curve.

### 2.9 Western blot

Western blot was performed as previously described.^16^ The right brain hemispheres were homogenized in RIPA (Santa Cruz Biotechnology) and centrifuged at 14,000 *g* at 4°C for 30 min. Equal samples were loaded on an SDS‐PAGE gel, and then the proteins were electrophoresed and transferred to a nitrocellulose membrane. The membranes were incubated overnight at 4°C with the primary antibodies as followed: rabbit anti‐TREM‐1 (1:1000, Abcam), rabbit anti‐SYK (1:1000, Abcam), rabbit anti‐β‐Catenin (1:3000, Abcam), rabbit anti‐phospho‐β‐Catenin (1:1000, Cell Signaling Technology), rat anti‐ ZO‐1 (1:500, Santa Cruz Biotechnology), rabbit anti‐Claudin‐5 (1:1000, Abcam), and mouse anti‐β‐actin (1:3000, Santa Cruz Biotechnology). The respective secondary antibody (1:3000, Santa Cruz Biotechnology) were incubated at room temperature for 2 h. Immunoblots were probed with an ECL Plus chemiluminescence reagent kit (Amersham Biosciences) and visualized with an imaging system (Bio‐Rad, VersaDoc, model 4000). Relative density was analyzed using Image J software.

### 2.10 Immunofluorescence staining

Mice were deeply anesthetized and perfused with cold PBS followed by 10% formalin transcardially. Brains were removed and fixed in formalin at 4°C overnight and then dehydrated at 10%–30% sucrose over 3 days. The brains were cut into 10‐μm‐thick with a cryostat (Leica Microsystems, CM3050S). Double fluorescence staining was performed as described previously.^17^ Primary antibodies were used as follows: rat anti‐TREM‐1 (1:100, Thermo Fisher Scientific), mouse anti‐vWF (1:100, Santa Cruz Biotechnology) and goat anti‐GFAP (1:200, Abcam).

### 2.11 Nissl staining

In long‐term assessment, mice were sacrificed at 28 days after ICH. Brains were fixed in formalin, dehydrated at 10%–30% sucrose, and sectioned at 20 μm. Sections were processed for Nissl staining to evaluate neuronal degeneration as previously described.^18^ The number of surviving neurons was counted in the hippocampal subfield (including CA1, CA3 and DG) under a light microscope.

### 2.12 Statistical analysis

Data were shown as the mean and standard deviation (mean ± SD). Statistical analysis was performed with GraphPad Prism 6.0. The Kolmogorov–Smirnov test was applied to check the normal distribution of the data, while F‐test was used to check the homogeneity of variances. Multiple comparisons were statistically analyzed using one‐way analysis of variance (ANOVA) followed by Tukey's HSD post hoc test. Long‐term neurobehavioral results were analyzed by two‐way ANOVA. *p* < 0.05 was considered statistically significant.

## 3 RESULTS

### 3.1 ICH increased the expression of TREM‐1 in the neurovascular unit

**FIGURE 1:**
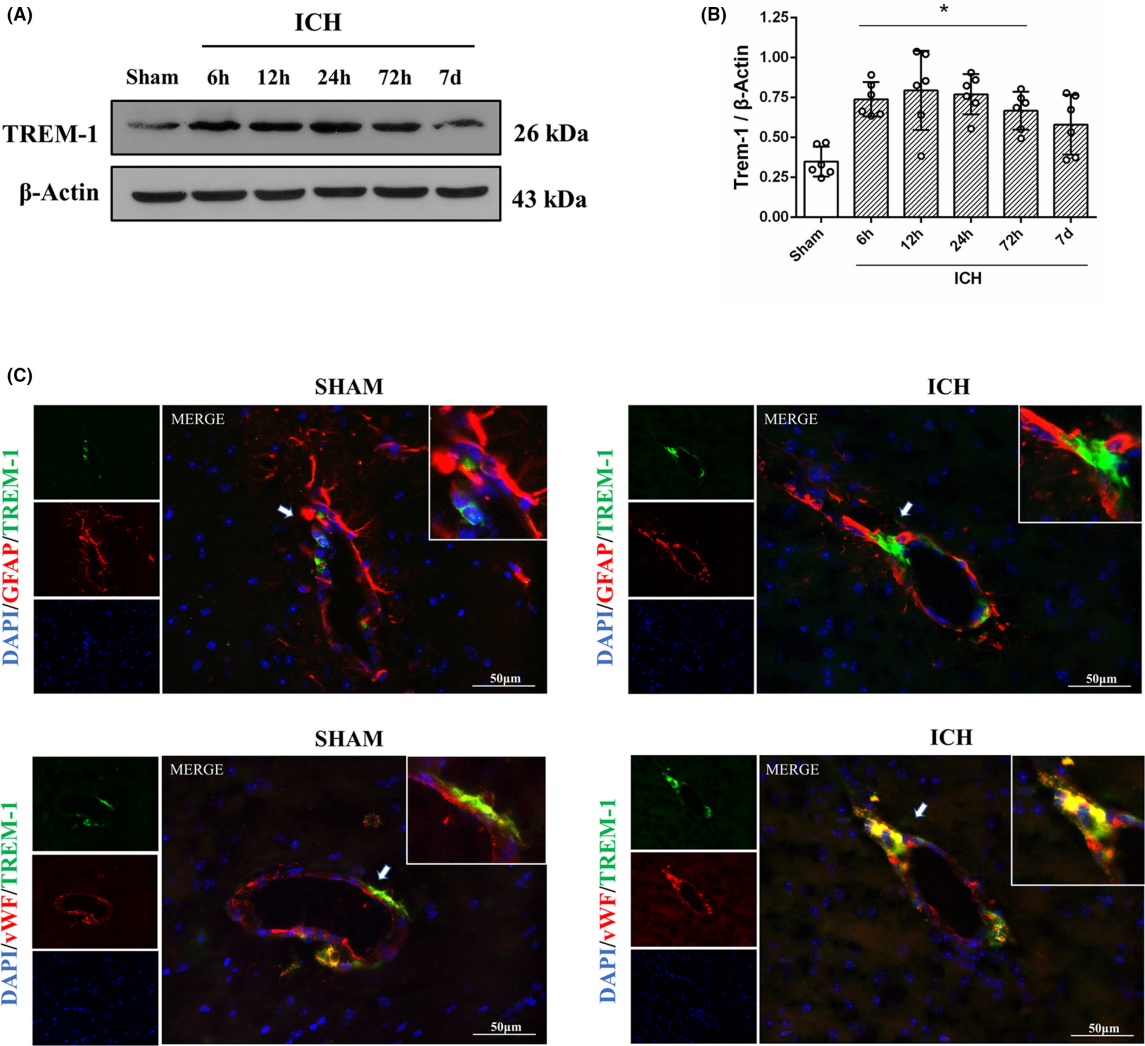
Temporal expressions of TREM‐1 and its neurovascular unit cell location after ICH. (A, B) Immunoblots and Quantitative analysis of TREM‐1. The error bars represent the mean ± SD. *p < 0.05 versus sham. *N* = 6 per group. (C) Double immunofluorescence staining for TREM‐1 (green) with astrocytes (GFAP, red) and endothelial cells (vWF, red) around the hematoma at 24 h after ICH. *N* = 2 per group. Scale bar, 50 μm. DAPI, 4′,6‐diamidino2‐phenylindole; GFAP, glial fibrillary acidic protein; vWF, von Willebrand factor.

The temporal expression of the TREM‐1 receptor after ICH was evaluated by western blot. The result showed that the expression of TREM‐1 significantly upregulated surrounding hematoma, which started at 6 h and extended to 7 days after ICH (*p* < 0.05, Figure 1A,B). Furthermore, double immunofluorescence staining was performed to determine the cellular localization of TREM‐1 in the neurovascular unit after ICH. As shown in Figure 1, TREM‐1 is mainly expressed on the endotheliocytes and almost not on astrocytes.

### 3.2 LP17 improved neurobehavioral outcomes and decreased brain edema after ICH

**FIGURE 2:**
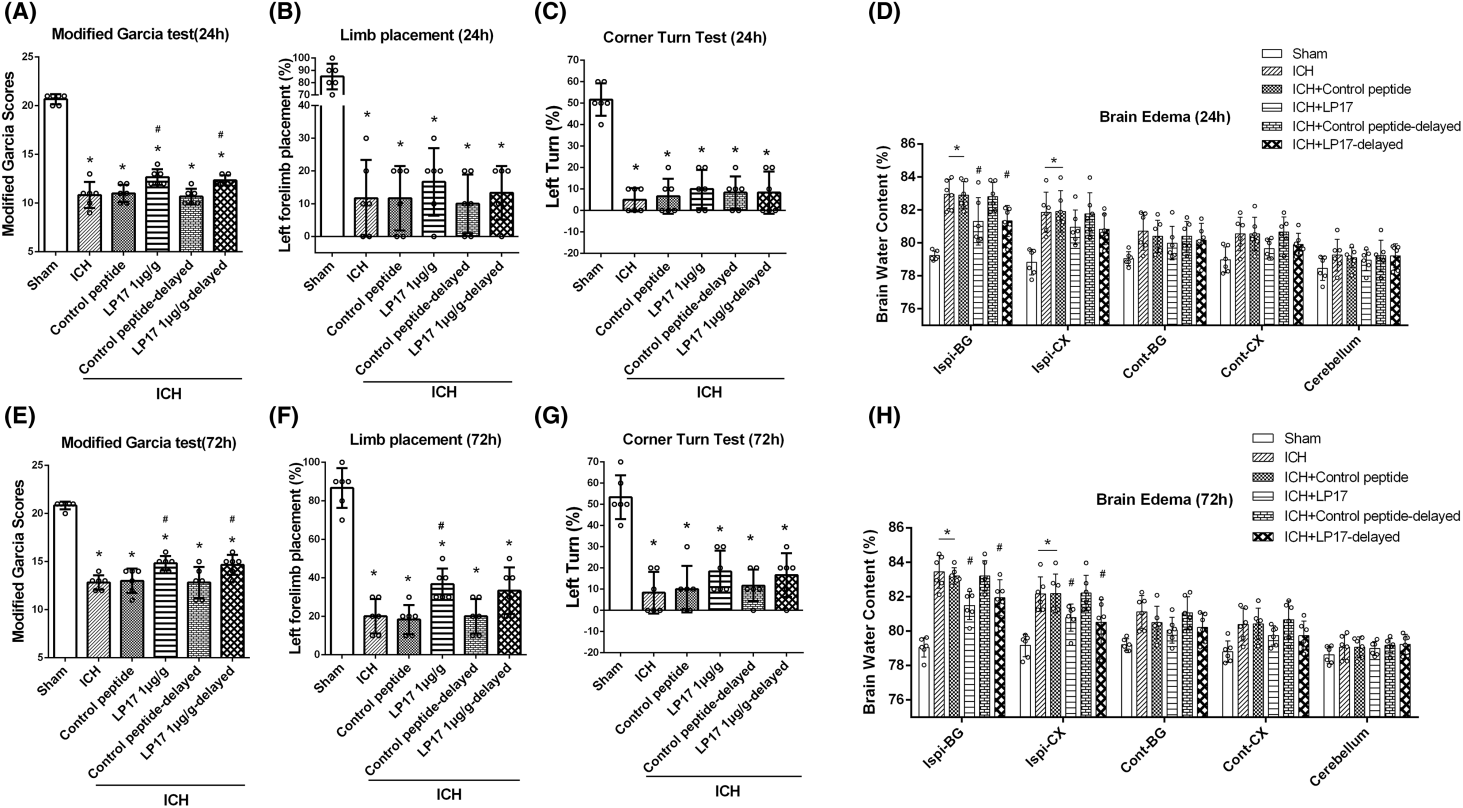
LP17 improved neurobehavioral deficits and decreased brain edema at 24 and 72 h after ICH. (A) modified Garcia test, (B) limb placement test and (C) corner turn test 24 h after ICH. (D) Analysis of brain water content in different brain regions at 24 h after ICH. (E–H) Result of neurobehavioral test and brain water content at 72 h after ICH. The error bars represent the mean ± SD. *p < 0.05 versus sham, ^#^ *p* < 0.05 versus ICH. *N* = 6 per group. BG, basal ganglia; Cont, contralateral; CX, cortex; Ispi, ipsilateral.

As previously described, a selective inhibitor of TREM‐1, LP17, was administrated intranasally at 2 h after ICH.^11^ Three types of neurobehavior tests and brain water content were evaluated at 24 and 72 h after ICH. As expected, ICH mice performed worse than sham mice in all neurobehavior tests, including the modified Garcia test (10.8 ± 1.3 vs. 20.7 ± 0.5, *p* < 0.05, in 24 h; 12.8 ± 0.8 vs. 20.8 ± 0.4, *p* < 0.05, in 72 h), limb placement test (11.6 ± 11.6 vs. 85.0 ± 10.5, *p* < 0.05, in 24 h; 20.0 ± 8.9 vs. 86.7 ± 10.3, *p* < 0.05, in 72 h), and corner turn test (5 ± 5.5 vs. 51.7 ± 7.5, *p* < 0.05, in 24 h; 8.3 ± 9.8 vs. 53.3 ± 10.3, *p* < 0.05, in 72 h). No significant difference between ICH and ICH plus control peptide groups were found at 24 h after ICH. Compared with ICH mice, 1.0 μg/g LP17 treated mice had higher scores in the modified Garcia test (12.7 ± 0.8 vs. 10.8 ± 1.3, *p* < 0.05) but no improvement in the other two tests (Figure 2A–C). And similar results of neurobehavioral tests were found at 72 h after ICH (14.8 ± 0.8 vs. 12.8 ± 0.8, *p* < 0.05, Figure 2E–G). Furthermore, delayed administration of LP17 also improved neurobehavioral outcomes in modified Garcia test at 24 and 72 h after ICH (12.3 ± 0.5 vs. 10.8 ± 1.3, *p* < 0.05, in 24 h; 14.7 ± 1.0 vs. 12.8 ± 0.8, *p* < 0.05, in 72 h). Meanwhile, LP17 significantly decreased the brain water content in basal ganglia of the ipsilateral hemisphere at 24 and 72 h after ICH (*p* < 0.05, Figure 2D,H).

### 3.3 LP17 preserved the blood–brain barrier permeability after ICH

**FIGURE 3:**
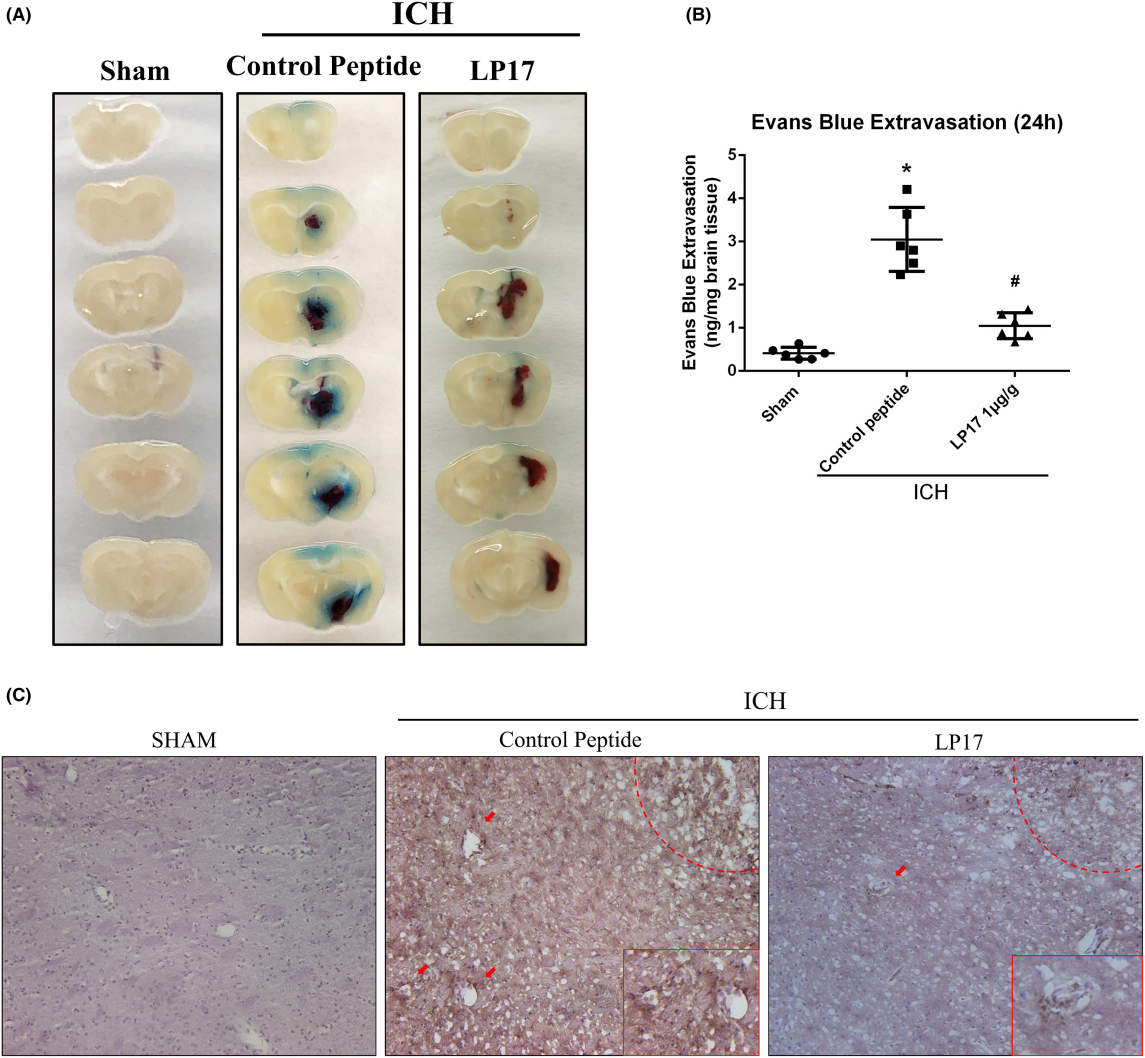
Inhibition of TREM‐1 reduced BBB leakage 72 h after ICH. (A) Pictures of brain slices 2 mm thickness with extravasated EB dyes in different groups. (B) Statistical analysis of EB extravasation. The error bars represent the mean ± SD, *p < 0.05 versus sham, ^#^ *p* < 0.05 versus ICH + Control peptide. *N* = 6 per group. (C) Immunohistochemistry for perivascular IgG staining surrounding hematoma.

Evans blue extravasation assay was measured at 72 h after ICH to assess the effect of TREM‐1 on the BBB permeability. EB extravasation in the right hemisphere increased significantly after ICH, while LP17 treatment obviously reduced EB leakage compared with the control peptide group (*p* < 0.05, Figure 3A,B). To further evaluate the BBB permeability after LP17 treatment, IgG protein staining with immunohistochemistry was used. As shown in Figure 3C and Appendix S1, the LP17 treatment group had a lower perivascular IgG protein leakage when compared with ICH plus control peptide group.

### 3.4 LP17 improved the long‐term neurobehavioral outcome after ICH

**FIGURE 4:**
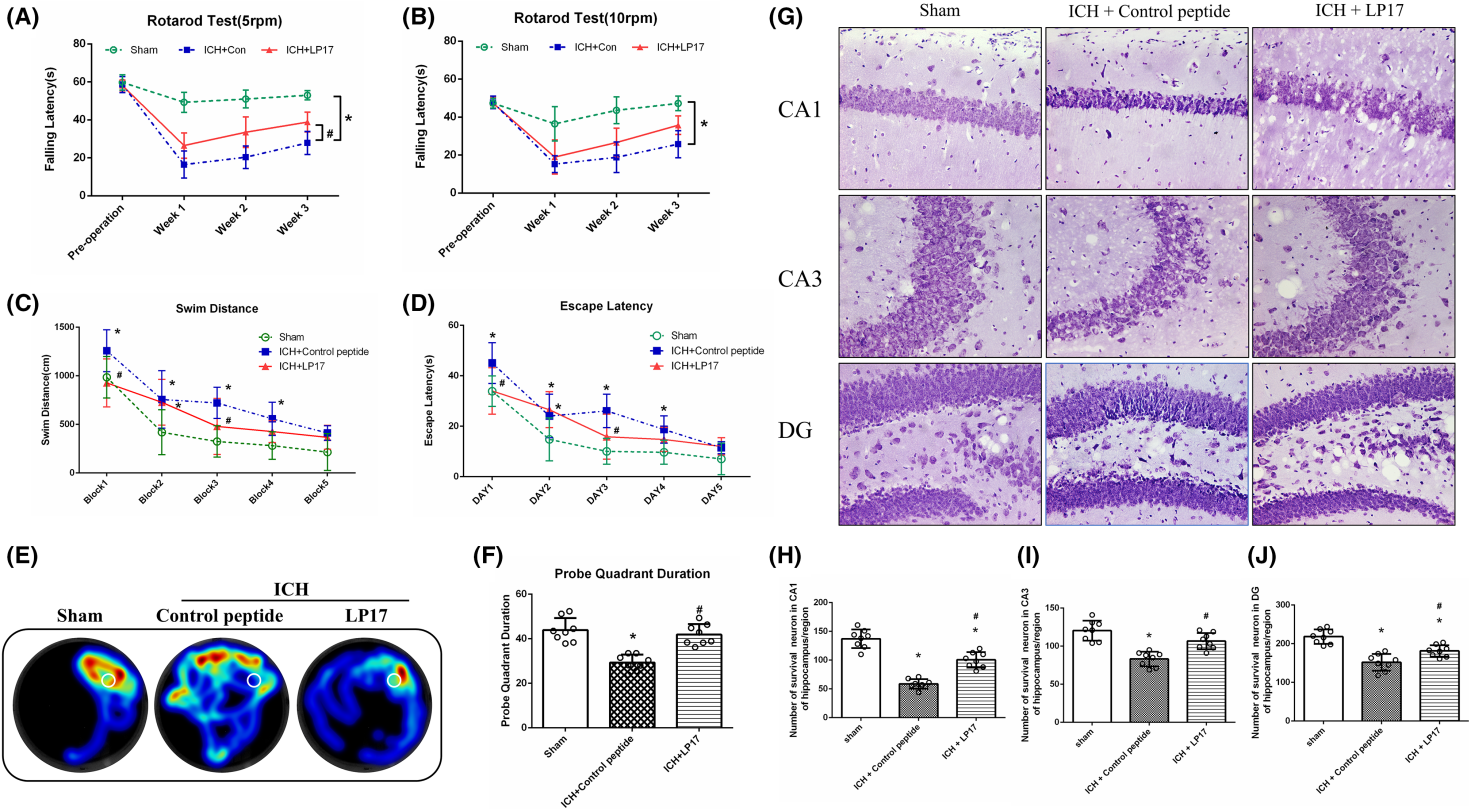
The effect of LP17 on the long‐term neurobehavioral function and neuron survival after ICH. (A, B) Rotarod test before operation and every week after ICH. (C, D) Swimming distance and escape latency of Morris water maze test on days 23–27 after ICH. (E) Representative heat map in probe trial at day 28. The white circles indicate the positions of the probe platform. (F) Probe quadrant duration. (G) Representative Nissl staining figures in difference regions of hippocampus including CA1, CA3 and DG. (H–J) Quantifications of survival neuron in CA1 (H), CA3 (I) and DG (J). The error bars represent the mean ± SD, *p < 0.05 versus sham, ^#^ *p* < 0.05 versus ICH, *N* = 8 per group.

Rotarod test and Morris water maze were applied to assess the effect of LP17 on long‐term neurobehavior. The Rotarod test showed a shortened falling latency in the control group at 5 and 10 rpm accelerating velocity tests compared with the sham group. And LP17 significantly increased falling latency at 5 rpm in 1–3 weeks after ICH (*p* < 0.05, Figure 4A,B). In the Morris water maze, mice in the control group had a significantly longer swim distance and time of escape latency to find the platform than sham mice. However, LP17 improved the performance on days 1 and 3 (Figure 4C,D), and LP17 treatment also increased the time spent in the probe quadrant trials in ICH mice (Figure 4F). and the result suggested that LP17 spatial memory and learning ability of ICH mice on long‐term.

Furthermore, Nissl staining was used to evaluate the survival of neurons in the hippocampus at 28 days after ICH. As shown in Figure 4, the survival of neurons in the CA1 region of the right hippocampus from the control group significantly decreased compared with the sham. And there was less neuron loss and shrinkage neuron in the LP17 treatment group. Meanwhile, similar results were found in the CA3 and DG regions of the hippocampus in three groups (Figure 4G–J).

### 3.5 TREM‐1 regulated the expression of junction molecules via the SYK/β‐catenin signaling pathway

**FIGURE 5:**
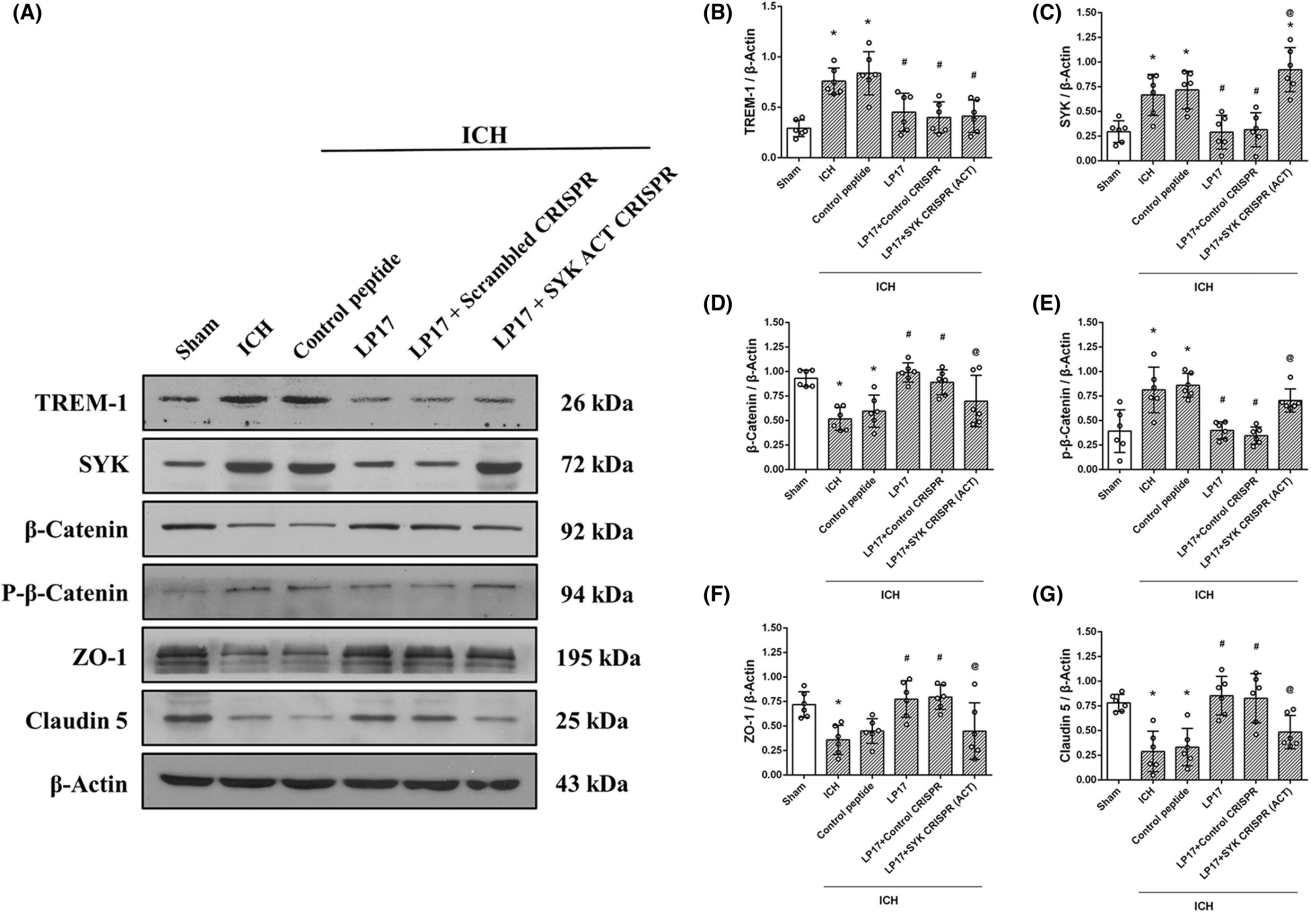
SYK activation CRISPR abolished the effect of LP17 at 24 h after ICH. (A) Representative Western blot bands. (B–G) Quantitative analyses of TREM‐1, SYK, β‐Catenin, p‐β‐Catenin, ZO‐1 and Claudin 5 in the ipsilateral hemisphere at 24 h after ICH. The error bars represent the mean ± SD. *p < 0.05 versus sham, ^#^ *p* < 0.05 versus ICH, ^@^ *p* < 0.05 versus LP17, One‐way ANOVA, Tukey's test. *N* = 6 per group.

To further explore the intracellular signal of TREM‐1 that regulated the expression of junction molecules, an SYK activation (ACT) CRISPR was applied to activate the predicted pathway before LP17 administration. As expected, the level of SYK was increased after ICH, accompanied with a downregulation of β‐catenin and upregulation of p‐β‐catenin, which indicated that SYK may participate in the phosphorylation of β‐catenin and influence the expression of junction molecules. LP17 treatment effectively decreased the expression of SYK and further attenuated β‐catenin phosphorylation. However, SYK ACT CRISPR significantly reversed the effect of LP17 on the phosphorylation of β‐catenin and the expression of junction molecules, which led to a downregulation of ZO‐1 and Claudin‐5 (Figure 5B–G).

**FIGURE 6:**
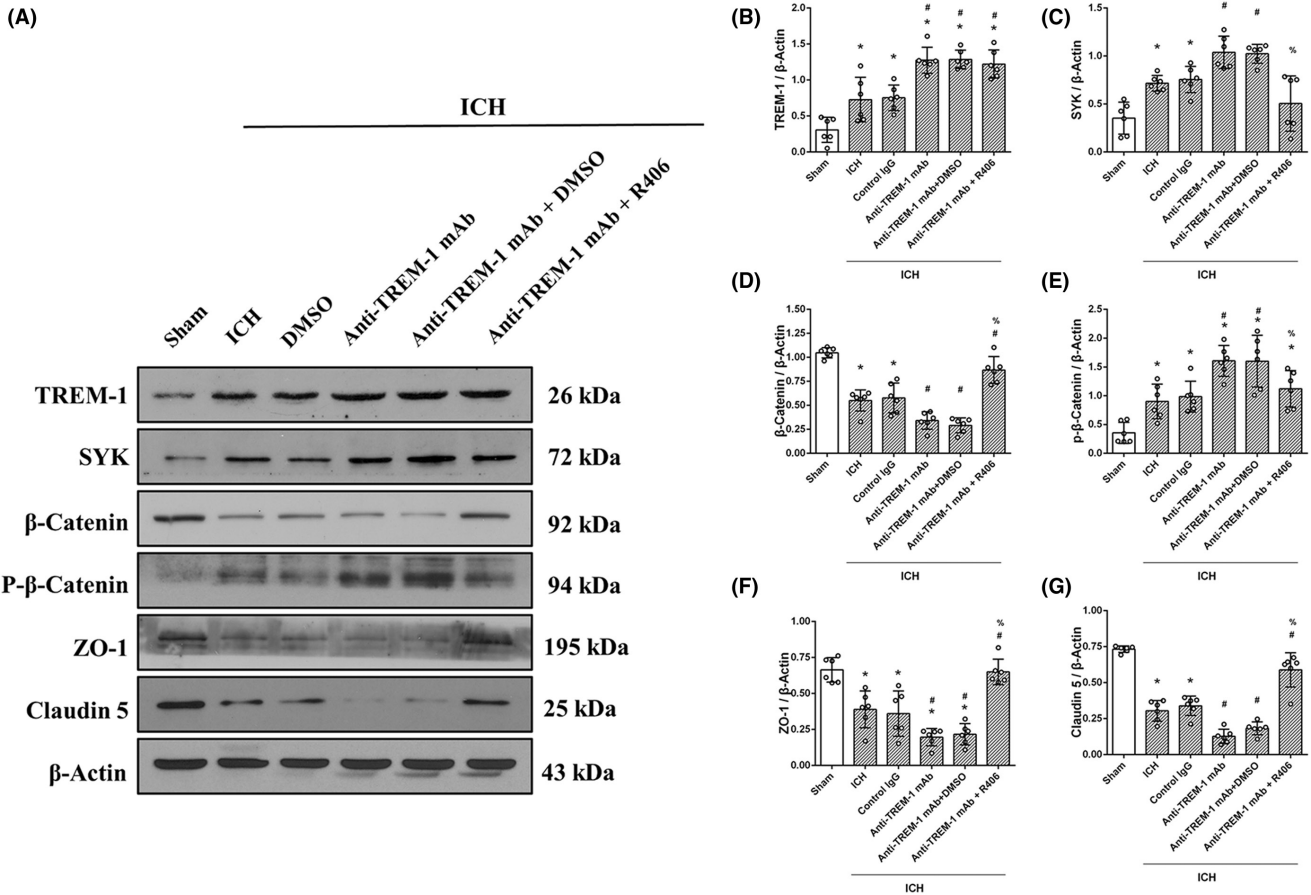
R406 reversed the effect of TREM‐1 activator on the expression of adhesion molecules at 24 h after ICH. (A) Representative Western blot bands. (B–G) Quantitative analyses of TREM‐1, SYK, β‐Catenin, p‐β‐Catenin, ZO‐1 and Claudin 5 in the ipsilateral hemisphere at 24 h after ICH. The error bars represent the mean ± SD. *p < 0.05 versus sham, ^#^ *p* < 0.05 versus ICH, ^%^ *p* < 0.05 versus Anti‐TREM‐1 mAb, One‐way ANOVA, Tukey's test. *N* = 6 per group.

Furthermore, we also evaluate the effect of the TREM‐1 activator on the expression of junction molecules using Anti‐TREM‐1 mAb before ICH. We found that activation of the TREM‐1 receptor decreased the level of junction molecules via the SYK/β‐catenin signaling pathway. Meanwhile, a selective inhibitor of SYK, called R406, was injected after ICH to reverse the signaling pathway. As expected, the phosphorylation of β‐catenin and the expression of junction molecules were reversed when compared to the Anti‐TREM‐1 mAb group (Figure 6).

## 4 DISCUSSION

In the present study, the following novel findings were made: (1) TREM‐1 receptor activated rapidly in collagenase‐induced ICH, which is similar to our previous study^11^ and is mainly expressed on the endotheliocytes in the vascular endothelial unit. (2) A selective inhibitor of TREM‐1, called LP17, decreased brain edema and improved neurobehavioral outcomes at 24 and 72 h after ICH, with a similar result in delayed LP17 treatment. (3) TREM‐1 receptor modulated the blood–brain barrier integrity after ICH via SYK/ β‐catenin signaling.

The blood–brain barrier is mainly composed of cerebral endothelial cells and interendothelial tight junctions. Under physiological conditions, the tightness of junction molecules contributes to a very low permeability of BBB unless some lipophilic or transported compounds, which provide the central nervous system (CNS) a highly controlled and healthy microenvironment.^19^ Numbers of studies verify that disrupted BBB integrity and increased permeability subsequently are critical aspects inducing secondary brain injury after ICH.^20^ BBB injury will result in the formation of brain edema, the neuroinflammation caused by leukocyte facilitation, and the entry of some harmful molecules.^21^ Thus, maintaining BBB integrity and permeability is a potential target for ICH.

Triggering receptor expressed on myeloid cells 1 usually expresses on the majority of innate immune cells, and it will amplify the inflammatory response when activated. Our previous study suggested that the TREM‐1 receptor activated rapidly on the surface of microglia after ICH and participated in the polarization of microglia. Furthermore, inhibition of TREM‐1 with LP17 could improve neurobehaviour outcomes and brain edema. Brain edema is related to the broken blood–brain barrier directly. To further investigate the role of TREM‐1 in the blood–brain barrier integrity, we conducted this study. As a result, we found TREM‐1 expressed on the endothelial cells in a collagenase‐induced ICH mouse and modulated the BBB permeability via regulating the expression of interendothelial adhesion molecules. Consistent with our result, the level of TREM‐1 expression was increased in microglia and endothelial cells in subarachnoid hemorrhage rats. It may aggravate early brain injury by interacting with the TLR4 pathway.^22^ However, the relation between the TREM‐1 receptor and BBB permeability had not been dissected. In our study, EB dye extravasation assay and IgG staining showed an increasing BBB permeability surrounding hematoma in ICH mice as reported before.^23^ LP17 could reduce the leakage of dye and IgG from microvascular in the brain via inhibiting TREM‐1 activation. Our study indicated that TREM‐1 activated in the endothelial cells and deteriorated BBB permeability after ICH, which may lead to augmenting the extent of brain edema.

LP17 is a synthetic peptide with complementary determining region‐3 and the “F” β strand of the extracellular domain of TREM‐1. LP17 could impair the TREM‐1 dimerization and compete with the natural ligand, then inhibit the bioactivity of TREM‐1.^24^ Based on the result of our previous study and other studies published,^25^ 1.0 μg/g LP17 was delivered intranasally at 2 h after ICH. Similar to auto‐blood‐induced ICH, LP17 also improves neurobehaviour outcomes and reduces brain edema in collagenase‐induced ICH mice. Considering that ICH patients may not get treatment in 2 h, LP17 was administrated at 6 h in this study, and the results suggest the equivalent benefits in delayed therapy.

Another major finding in this study was that the TREM‐1 receptor regulated the expression of junction molecules via the SYK/β‐catenin signaling pathway. As reported, TREM‐1 features with a cytoplasmic domain called DAP12 (DNAX activating protein of 12 kDa), and DAP12 will recruit and activate SYK when TREM‐1 is activated.^4^ In breast cancer, SYK was involved in the maintenance of the epithelial integrity of the gland via regulating the phosphorylation of β‐catenin and stabilizing the E‐cadherin adherens junction complex.^26^ Meanwhile, endothelial β‐catenin is an indispensable intracellular mediator maintaining BBB integrity, and phosphorylation of β‐catenin, as a form of degeneration, will induce downregulation of interendothelial junctions and result in BBB breakdown.^27^ However, the relation between SYK and β‐catenin after ICH is poorly characterized. In our study, LP17 administration significantly downregulated the expression of SYK, upregulated the expression of β‐catenin, and increased the level of interendothelial junction molecules, including ZO‐1 and Claudin‐5. SYK ACT CRISPR reversed those effects of LP17. The results indicate that TREM‐1 regulated the expression of junction molecules via the SYK/β‐catenin signaling pathway. Furthermore, we applied Anti‐TREM‐1 mAb and R406 to prove our idea.

There are several limitations in the present study. First, TREM‐1 is also expressed in the microglia as we reported.^11^ Thus, the role of neuroinflammation needs to be considered. Second, we point out SYK/β‐catenin signaling as a critical downstream of TREM‐1 that modulates BBB permeability. However, they may not be the only signaling pathway of TREM‐1, and TREM‐1 may regulate the BBB permeability through other proteins. Third, using only male and adult animals may cause some bias. Furthermore, LP17 may leak out of the brain due to the increased BBB permeability. More studies need to demonstrate the retention of LP17 in the brain. Last but not least, this study did not conduct cellular experiments, which may help confirm the role of TREM‐1 in endothelial cells.

## 5 CONCLUSION

In summary, our study demonstrated that the TREM‐1 receptor increased brain edema by regulating blood–brain barrier permeability after intracerebral hemorrhage in mice. This effect was mediated by the SYK/β‐catenin signaling pathway to influence the expression of interendothelial junction molecules. Thus, inhibition of the TREM‐1 receptor by LP17 may be a therapeutic target for ICH progression.

## AUTHOR CONTRIBUTIONS

YX conducted experiments, analyzed data and drafted the manuscript. WH conducted experiments and analyzed data. LM edited the manuscript. RR and WH conducted ICH model and neurobehavioral tests. SY and QL worked on designing the study and preparing manuscript.

## FUNDING INFORMATION

This work was supported by the Natural Science Foundation of Zhejiang Province, China (LQ21H090013), and the Medical Science and Health Technology of Zhejiang Province, China (2023KY786).

## CONFLICT OF INTEREST STATEMENT

All authors declare no competing interests.

## Supporting information


Appendix S1

## Data Availability

The data that support the findings of this study are available from the corresponding author upon reasonable request.
