# Supplementary material for: Endothelial TREM‐1 receptor regulates the blood–brain barrier integrity after intracerebral hemorrhage in mice via SYK/β‐catenin signaling
Source: CNS Neurosci Ther. 2023 May 11;29(11):3228–38. doi: 10.1111/cns.14255 (PMC10580358; doi:10.1111/cns.14255)
Supplement: Supplementary file 1 — Appendix S1 [file CNS-29-3228-s001.pdf]

Supplement in method

modified Garcia score

The modified Garcia score consists of seven items that evaluated spontaneous activity, axial sensation, vibrissae proprioception, symmetry of limb movement, lateral turning, forelimb walking, and climbing. And each item contains scores ranging from 0 to 3.

Forelimb placement test

The forelimb placement test was used to investigate the responsiveness of vibrissae stimulation for mice. When the mice approached the countertop's corner edge, the forelimb placing ipsilateral to the stimulated vibrissae was recorded. And results were recorded as a percentage of the number of successful left forelimb placements out of 10 consecutive stimulations.

Corner turn test

In the corner turn test, the mice were allowed to enter a 30° corner and exit by turning to the left or right. Left turning was recorded for ten trials, and the percentage of left turns in ten trials was calculated.

Rotarod test

The Rotarod test was conducted to evaluate sensorimotor coordination and balance in the first, second, and third weeks after ICH. The rotating speed started at 5 rpm and gradually accelerated by 2 rpm every 5 s. The duration that mice were able to stay on the accelerating rotating cylinder was recorded by a photobeam circuit.

Morris water maze

The Morris water maze was performed to assess the abilities of spatial learning and memory on days 21 to 25 after ICH. Each mice were performed a 6-day test and 5 trials per day. The mice were placed in a semi-random set of start locations to find a visible platform above the water level in 60 s. all animals' swimming paths, escape latency, and swim distance was recorded. No matter whether mice could find the platform or not, they were guided and allowed to stay on the platform for 5 s. On the last day, the platform was removed, and a computerized tracking system recorded the time mice took in the platform quadrant.

Supplementary tables and figures

Table. S1 Study groups and animal used

Experiment 1 and 5-1and 6-1	Numbers	Dead
Groups		
Sham	8	
ICH-6h	6	
ICH-12h	6	
ICH-24h	8	
ICH-72h	6+1	1
ICH-7d	6	
Experiment 2		
Groups-1		
Sham	6	
ICH	6	
ICH + Control peptide	6	
ICH + LP17 1μg/g	6	
ICH + Control peptide-delayed	6+1	1
ICH + LP17 1μg/g-delayed	6	
Groups-2		
Sham	6	
ICH	6	
ICH + Control peptide	6	
ICH + LP17 1μg/g	6	
ICH + Control peptide-delayed	6	
ICH + LP17 1μg/g-delayed	6	
Experiment 3		
Groups		
Sham	10	
ICH + Control peptide	10	
ICH + LP17 1μg/g	10	
Experiment 4		
Groups		
Sham	8	
ICH + Control peptide	8	
ICH + LP17 1μg/g	8	
Experiment 5		
Groups-1		

Sham	6	
ICH	6	
ICH + Control peptide	6	
ICH + LP17 1μg/g	6	
ICH + LP17 1μg/g + SYK Activation CRISPR	6+1	1
ICH + LP17 1μg/g + Control CRISPR	6	
Groups-2		
Sham	6	
ICH	6	
ICH + Control IgG	6	
ICH + Anti-TREM-1 mAb 0.25 μg/g	6+1	1
ICH + Anti-TREM-1 mAb 0.25 μg/g+ R406 5 μg/g	6	
ICH + Anti-TREM-1 mAb 0.25 μg/g + DMSO	6	
Total	242	4 (1.65%)

Fig. S1 Experimental design

Experiment 1: Time course and cellular localization of TREM-1

Groups:

Sham	n=8
ICH-6h	n=6
ICH-12h	n=6
ICH-24h	n=8
ICH-72h	n=6
ICH-7d	n=6

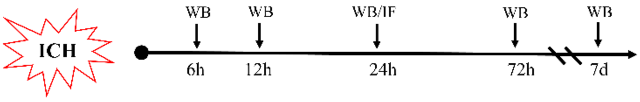

Experiment 2: Effect of TREM-1 on the outcome after collagenase induced ICH

Groups-1:

Sham	n=6
ICH	n=6
ICH + Control peptide	n=6
ICH + LP17 1 μg/g	n=6
ICH + Control peptide-delayed	n=6
ICH + LP17 1 μg/g delayed	n=6

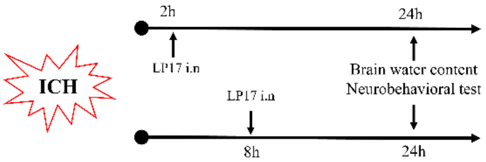

Groups-2:

Sham	n=6
ICH	n=6
ICH + Control peptide	n=6
ICH + LP17 1 μg/g	n=6
ICH + Control peptide-delayed	n=6
ICH + LP17 1 μg/g delayed	n=6

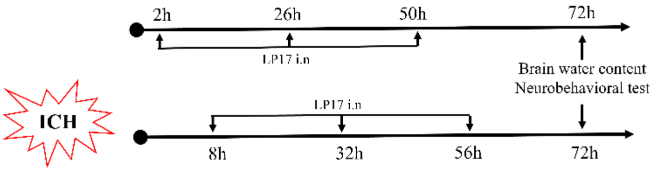

Experiment 3: Effect of TREM-1 on the permeability of blood-brain barrier

Groups:

Sham	n=10
ICH + Control peptide	n=10
ICH + LP17 1 μg/g	n=10

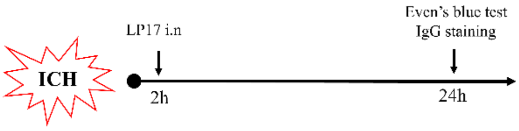

Experiment 4: Effect of TREM-1 on the long-term outcome after ICH

Groups:

Sham	n=8
ICH + Control peptide	n=8
ICH + LP17 1 μg/g	n=8

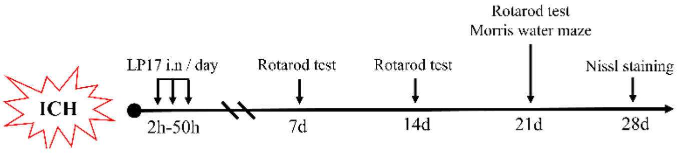

Experiment 5: TREM-1 regulates permeability of blood-brain barrier via SYK-β-catenin signaling pathway

Groups-1:

Sham	n=6
ICH	n=6
ICH + Control peptide	n=6
ICH + LP17 1 μg/g	n=6
ICH + LP17 1 μg/g + SYK Activation CRISPR	n=6
ICH + LP17 1 μg/g + Control CRISPR	n=6

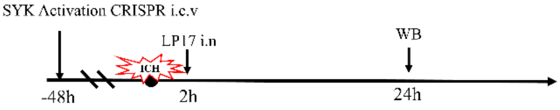

Groups-2:

Sham	n=6
ICH	n=6
ICH + Control IgG	n=6
ICH + Anti-TREM-1 mAb 0.25 μg/g	n=6
ICH + Anti-TREM-1 mAb 0.25 μg/g+ R406 5μg/g	n=6
ICH + Anti-TREM-1 mAb 0.25 μg/g + DMSO	n=6

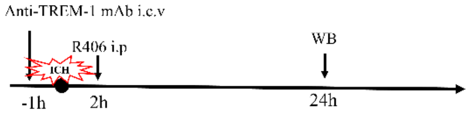

Fig. S2 Immunofluorescence for IgG staining

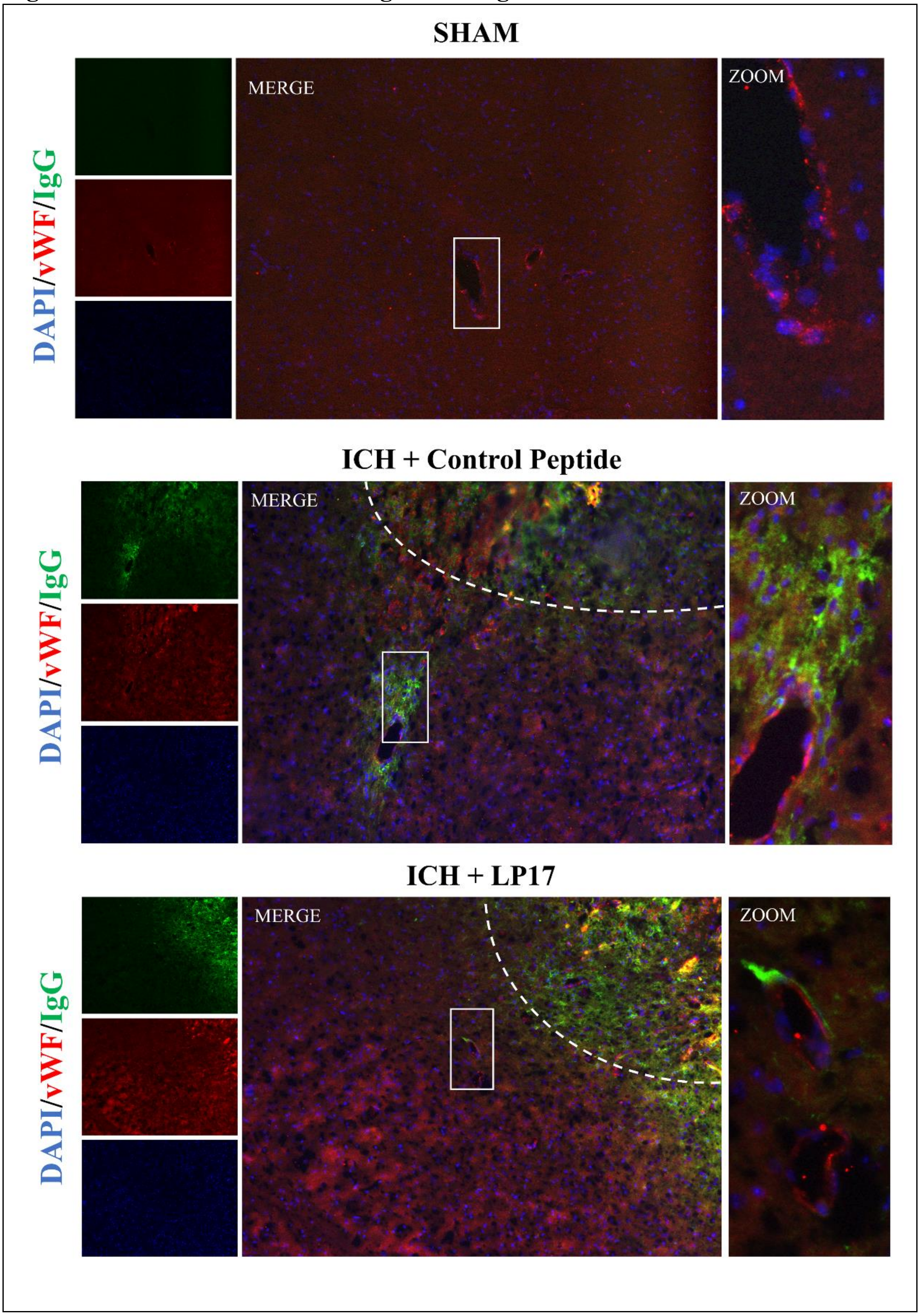

Fig. S2 LP17 reduced BBB leakage 72h after ICH. Representative image of immunofluorescence for perivascular IgG staining surrounding hematoma. vWF, von Willebrand factor; DAPI, 4′,6-diamidino2-phenylindole. The dotted line represents the hematoma margin. White boxes were magnified in "ZOOM."
